# Supplementary material for: Receptor for advanced glycation end-products (RAGE) mediates phagocytosis in nonprofessional phagocytes
Source: Commun Biol. 2022 Aug 16;5:824. doi: 10.1038/s42003-022-03791-1 (PMC9381800; doi:10.1038/s42003-022-03791-1)
Supplement: Supplementary file 2 — Supplementary Information [file 42003_2022_3791_MOESM2_ESM.pdf]

Supplementary Figure 1

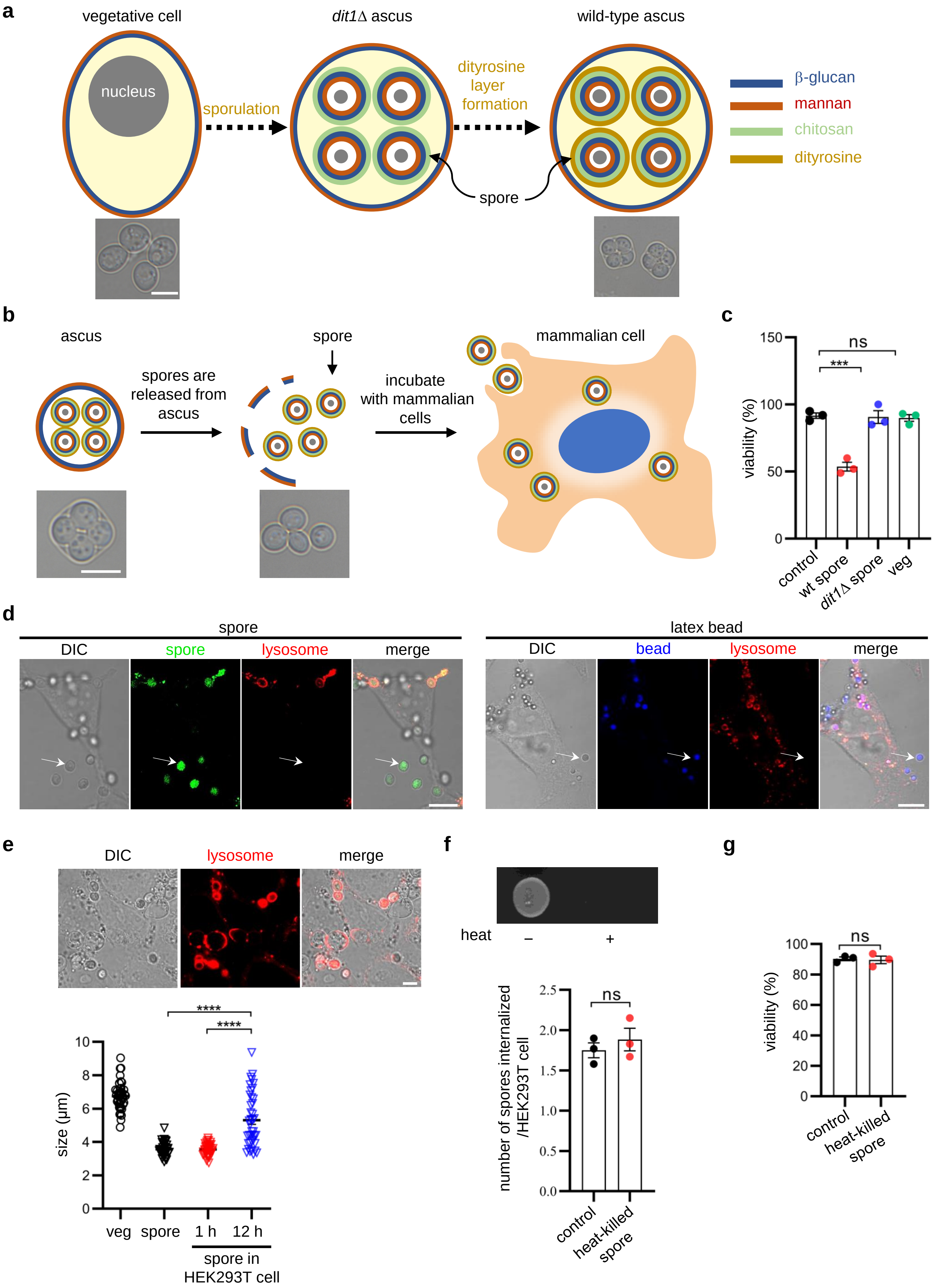

Supplementary Figure 1: Yeast spores exhibit cytotoxicity in HEK293T cells. a Schematic diagram of the spore formation process. Spores are formed in the cytosol of the mother cell. As a result of sporulation, the mother cell becomes an ascus, which includes four spores. The spore wall is composed of mannan, glucan, chitosan, and dityrosine. *dit1*Δ spores lack the dityrosine layer. Bottom panels are images of vegetative cells and asci. Scale bar, 5 µm. **b** Schematic diagram of the process to assess spore internalization in mammalian cells. Spores were released form asci by sonication and detergent treatment as described in Materials and Methods. Spores released form asci were incubated with mammalian cells in the assay. Bottom panels are images of an ascus and spores released from an ascus. Scale bar, 5 µm. **c** Cytotoxicity of yeast cells. HEK293T cells were incubated for 12 h with or without (control) vegetative cells (veg), wild-type (wt) spores, or *dit1*Δ spores at 1.2×10^7 yeast cells/4-6 $\times 10^5$ HEK293T cells/ml. Propidium iodide staining was used to detect dead cells. **d** Spores and latex beads in the culture medium are not stained by LysoTracker. HEK293T cells cultured in Dulbecco's modified Eagle's medium were incubated with spores or latex beads (tRNA-bound latex beads) at 1.2×10^7 spores or latex beads/4-6 $\times 10^5$ HEK293T cells/ml. After addition of LysoTracker Red, images were obtained with differential interference contrast (DIC) or fluorescence microscopy (lysosome). Arrows indicate the spore or latex bead that is not internalized in HEK293T cells. Scale bar, 10 µm. **e** Sizes of spores internalized in HEK293T cells. Upper panels: Representative images of swollen spores. Spores were incubated for 12 h with HEK293T cells. Images were obtained with differential interference contrast (DIC) or fluorescence microscopy (lysosome). Lysosomes were stained with LysoTracker Red. Scale bar, 5 µm. Lower panel: Distribution of size of spore internalized in HEK293T cells. Spores were incubated for 1 or 12 h with HEK293T cells. The size was determined by measuring the longest dimension of internalized spores. As controls, distribution of size of spore (spore) in sporulation media and vegetative cell (veg) in yeast growth media are shown. **f** Internalization of heat-killed spores in HEK293T cells. Upper panel: Viability assay of heat-killed spores. 3×10^7 of spores treated with (+) or without (-) heat (62℃, 60 min) were spotted onto YPAD plate to show spores were killed by the heat treatment. Lower panel: Spores (control) or heat-killed spores were incubated for 1 h with HEK293T cells. Mean numbers of spores internalized per cell are shown. **g** Cytotoxicity of heat-killed spores. HEK293T cells were incubated for 12 h with or without (control) heat-killed spores. Propidium iodide staining was used to detect dead cells. Data are presented as the mean ± SEM (**c,** and **e** to **g**). Statistical significance was determined by two-tailed unpaired Student's *t* tests. n=3 (**c**, **f**, and **g**). n=40 (**e**). ***, $P < 0.001$; ****, $P < 0.0001$; ns, not significant ($P \geq 0.05$).

Supplementary Figure 2

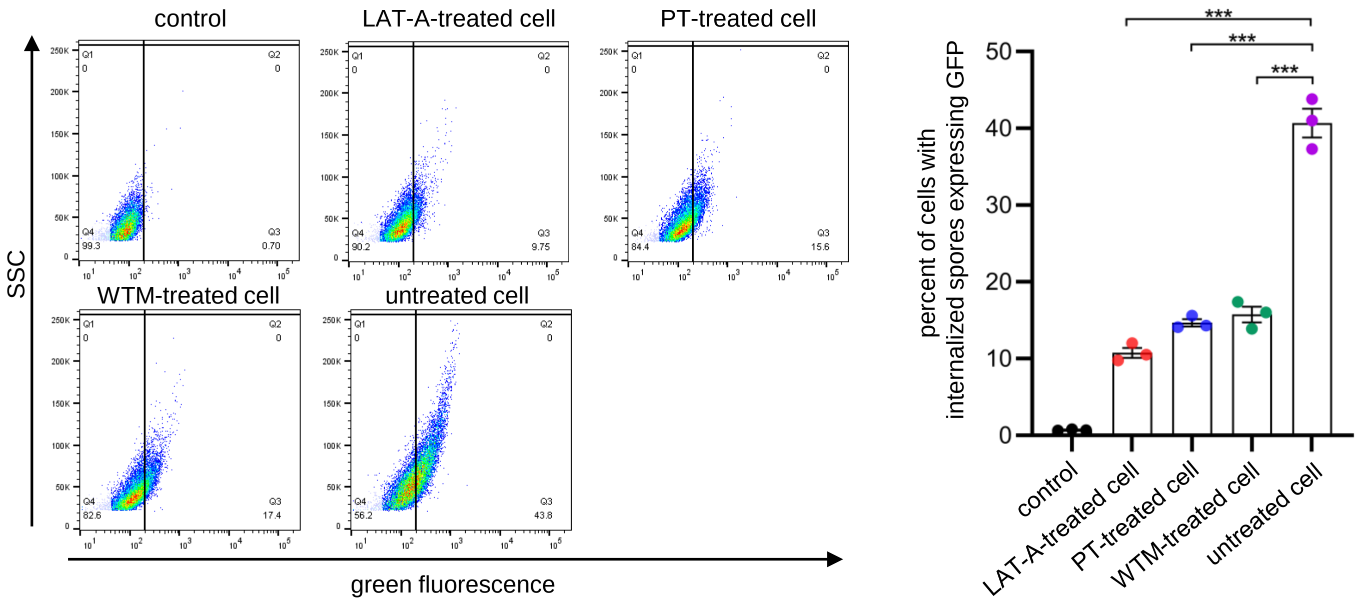

Supplementary Figure 2: Effect of pharmacological inhibitors on spore internalization by HEK293T cells using FACS. HEK293T cells were incubated with wortmannin (WTM), piceatannol (PT), or latrunculin A (LAT-A) for 30 min. The cells were incubated with spores expressing GFP at 1.2×10^7 spores/4-6 $\times 10^5$ HEK293T cells/ml. After 1 h incubation, HEK293T cells were subjected to FACS analysis. As a control, HEK293T cells without incubation with spores were subjected to FACS analysis. Left panels: Representative FACS dot plot images. Right panel: Quantification of HEK293T cells internalized spores. Data are presented as the mean $\pm$ SEM. Statistical significance was determined by two-tailed unpaired Student's *t* tests. *n*=3. ***, $P < 0.001$.

Supplementary Figure 3

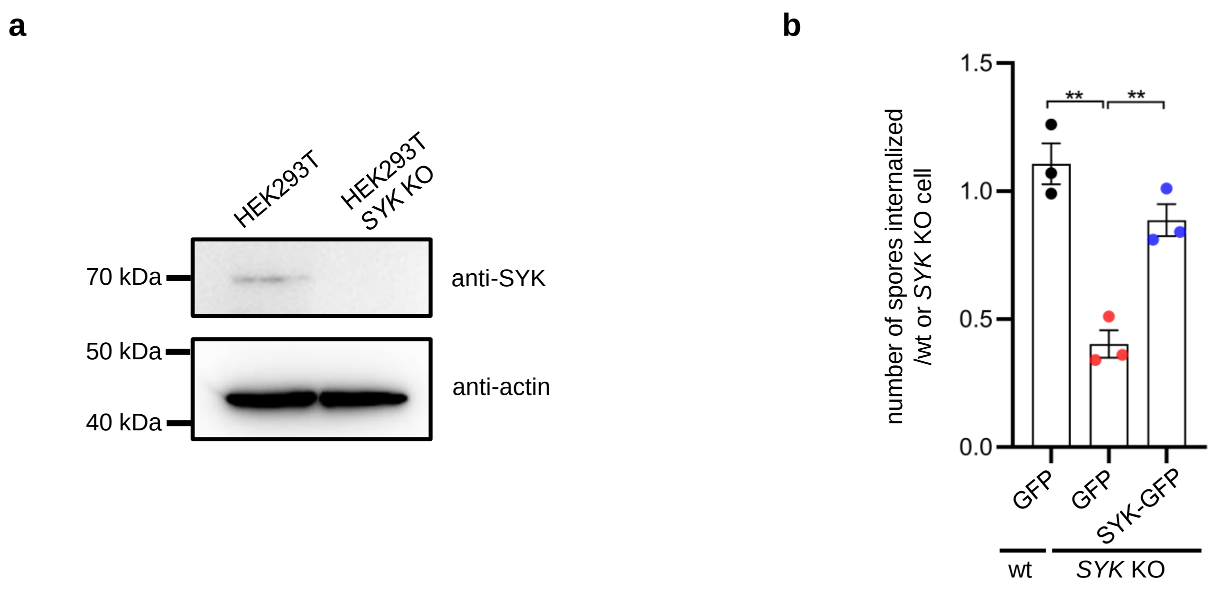

Supplementary Figure 3: SYK is required for internalization of spores in HEK293T cells. a Detection of SYK by western blotting analysis using an anti-SYK antibody in wild-type HEK293T and HEK293T *SYK* KO cell lysates. Actin is shown as a loading control. **b** Internalization of spores in HEK293T (wt) or HEK293T *SYK* KO cells expressing GFP alone or SYK fused to mEGFP (SYK-GFP) was analyzed. The cells were incubated with spores at 1.2×10^7 spores/4-6 $\times 10^5$ HEK293T cells/ml for 1 h. Data are presented as the mean $\pm$ SEM (**a**). Statistical significance was determined by two-tailed unpaired Student's *t* tests. n=3 (**b**). **, $P < 0.01$.

Supplementary Figure 4

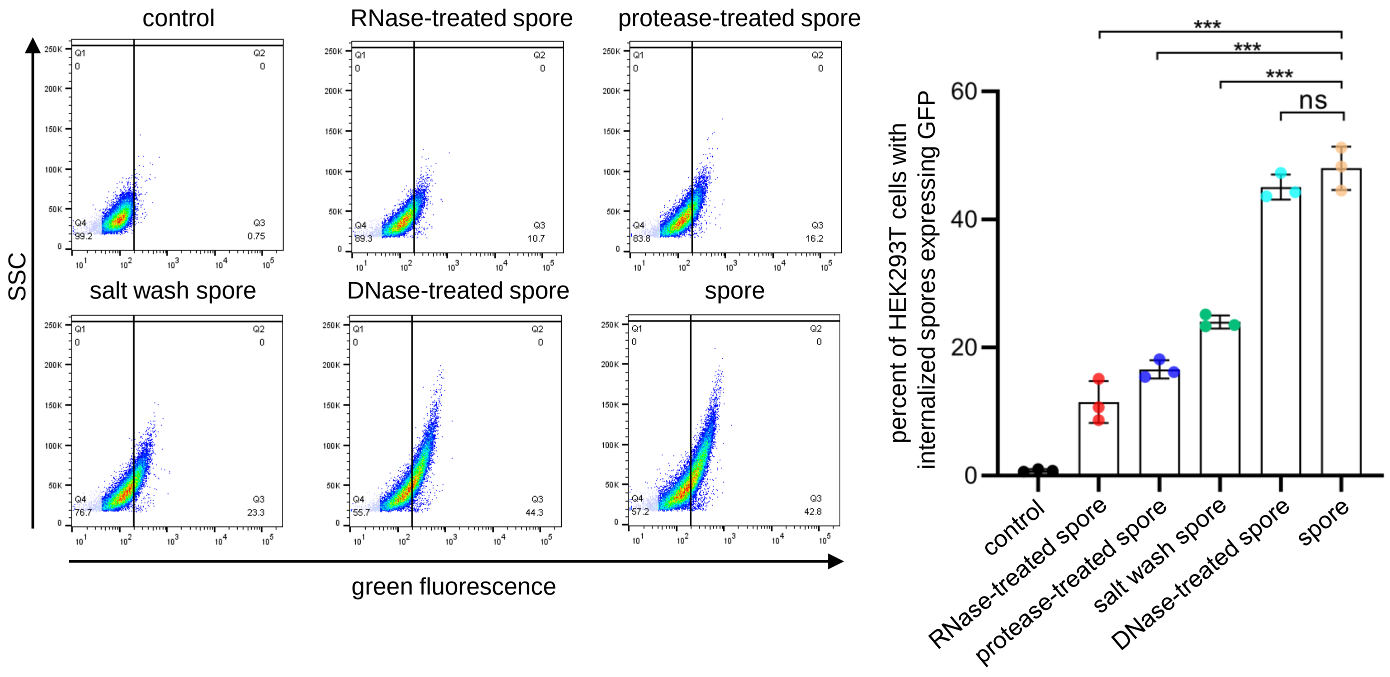

Supplementary Figure 4: Analysis of spore internalization by HEK293T cells using FACS. Spores expressing GFP were treated with indicated reagents and incubated with HEK293T cells at 1.2×10^7 spores/4-6 $\times 10^5$ HEK293T cells/ml. After 1 h incubation, HEK293T cells were subjected to FACS analysis. As a control, HEK293T cells without incubation with spores were subjected to FACS analysis. Left panels: Representative FACS dot plot images. Right panel: Quantification of HEK293T cells internalized spores. Data are presented as the mean $\pm$ SEM. Statistical significance was determined by two-tailed unpaired Student's *t* tests. *n*=3. ***, $P < 0.001$; ns, not significant ($P \geq 0.05$).

Supplementary Figure 5

a

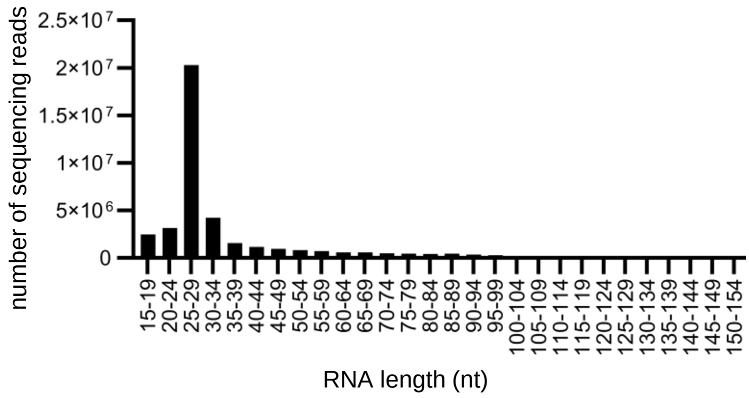

b

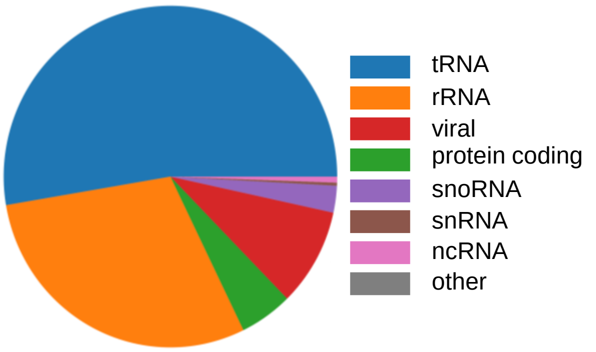

c

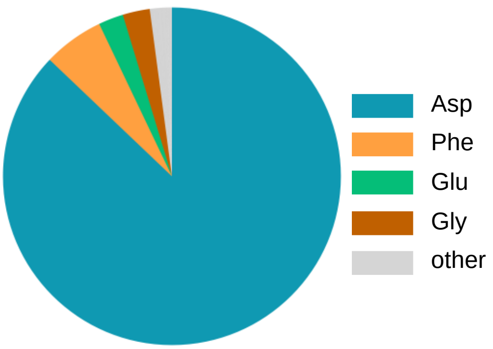

Supplementary Figure 5: Analysis of spore wall-bound RNA. a Distribution of RNA fragment length bound to the spore wall. Spore wall-bound RNA fragments eluted with 0.6 M NaCl solution were sequenced and distribution of RNA fragment length is shown. **b** The percent of reads mapping to the indicated RNA species. snoRNA, small nucleolar RNA; snRNA, small nuclear RNA; ncRNA, non-cording RNA. **c** The fraction of reads mapping to each of the indicated tRNA species.

Supplementary Figure 6

a

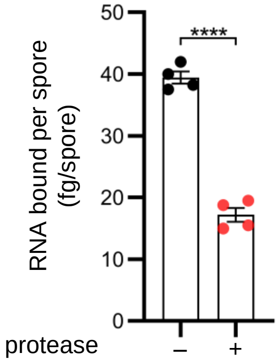

b

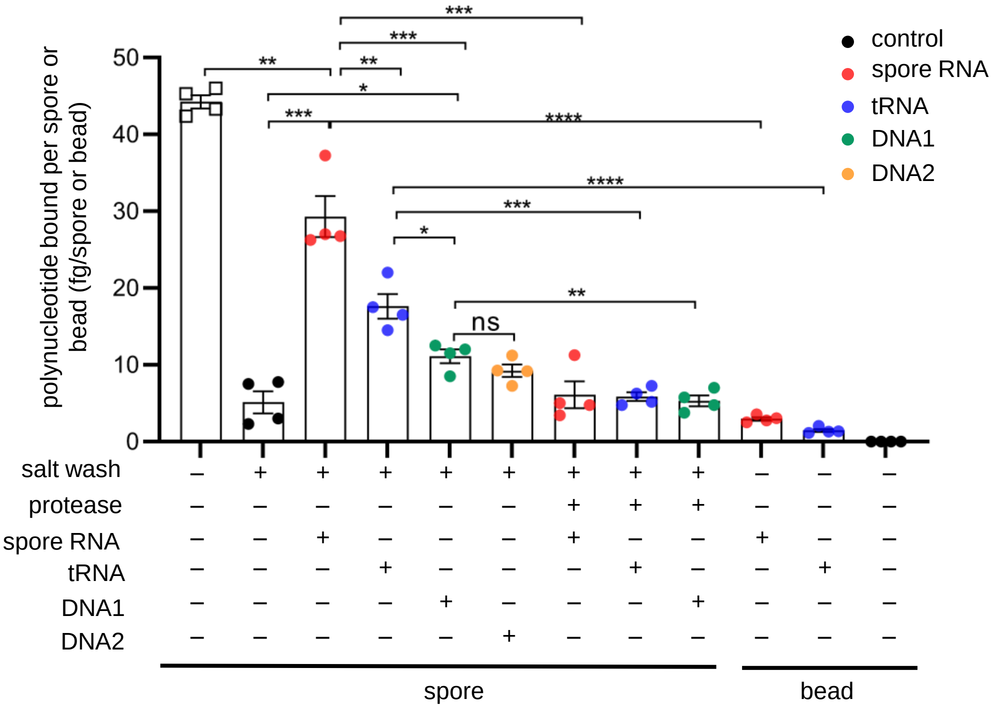

Supplementary Figure 6: Amounts of polynucleotides bound to latex beads or spores. a Amount of RNA purified from 2×10^8 spores treated with (+) or without (-) protease. **b** Amounts of polynucleotides bound to spore or latex beads. High-salt-washed spores were treated with (+) or without (-) protease. 2×10^8 spores were incubated with (+) or without (-) 40 µg of polynucleotides. As a control, RNA released from spores with high-salt is shown (salt wash -). Latex beads (2×10^8) were incubated with 200 µg of spore wall-derived RNA and tRNA. Polynucleotides bound to the spores or beads were eluted by high-salt solution and their amounts were measured. Data are presented as the mean ± SEM. Statistical significance was determined by two-tailed unpaired Student's *t* tests. *n*=4. *, $P < 0.05$; **, $P < 0.01$; ***, $P < 0.001$; ****, $P < 0.0001$; ns, not significant ($P \geq 0.05$).

Supplementary Figure 7

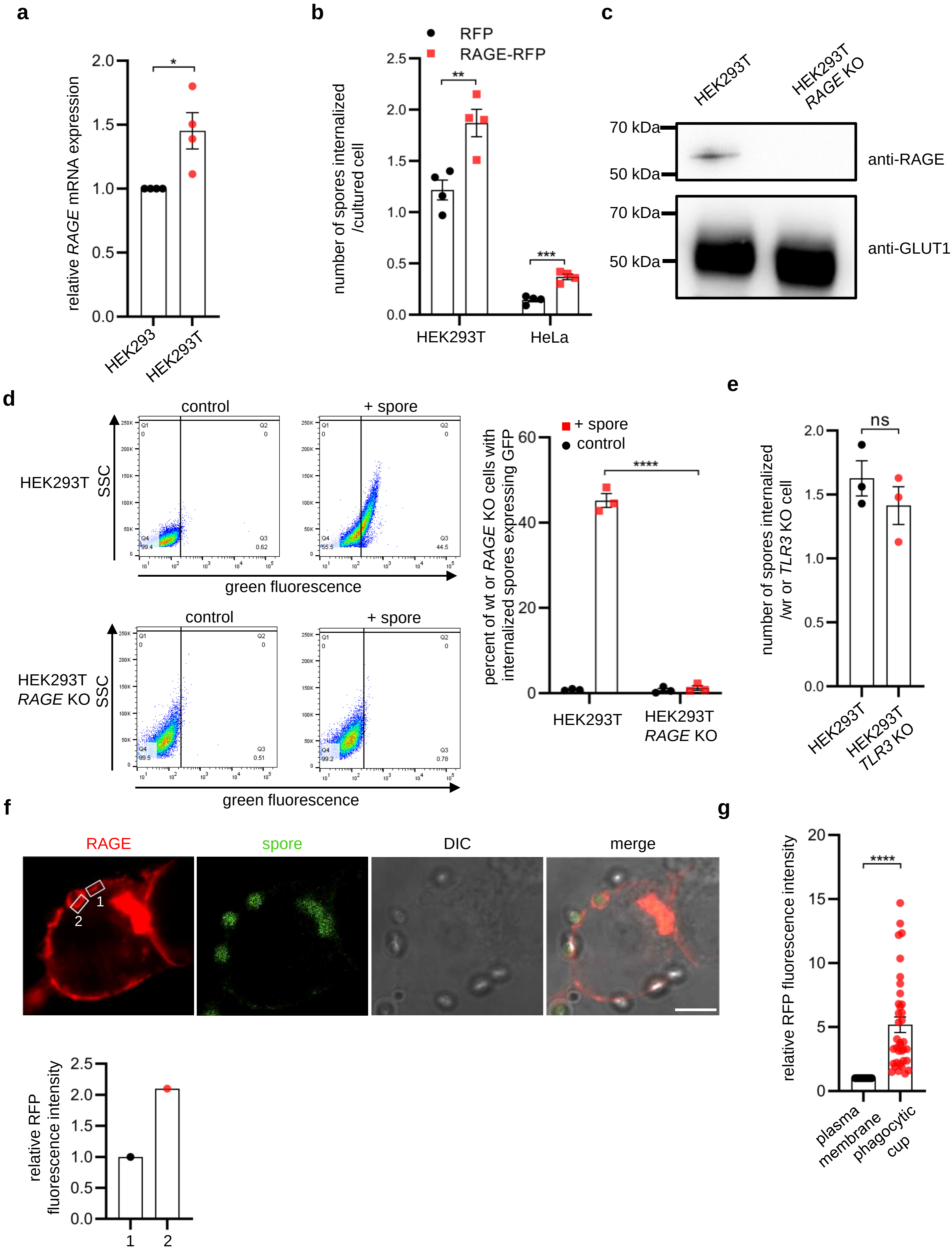

Supplementary Figure 7: RAGE is required for internalization of spores in HEK293T cells. a Levels of the *RAGE* mRNA in HEK293 cells and HEK293T cells. **b** Internalization of spores in HEK293T or HeLa cells transiently expressing RAGE-RFP or RFP alone. Mean numbers of spores internalized in cultured cell with RFP fluorescence are shown. **c** Detection of RAGE by western blotting analysis using an anti-RAGE antibody in wild-type HEK293T and HEK293T *RAGE* KO cell membrane fraction. Glucose transporter 1 (GLUT1) is shown as a loading control. **d** Spores expressing GFP were incubated with HEK293T or HEK293T *RAGE* KO cells at 1.2×10^7 spores/4-6 $\times 10^5$ cells/ml (+ spore). After 1 h incubation, the cells were subjected to FACS analysis. As a control, HEK293T or HEK293T *RAGE* KO cells without incubation with spores were subjected to FACS analysis. Left panels: Representative FACS dot plot images. Right panel: Quantification of HEK293T or HEK293T *RAGE* KO cells internalized spores. **e** Internalization of spores in HEK293T or HEK293T *TLR3* KO cells. **f** HEK293T cells were transiently transfected with RAGE-RFP. Cells were incubated with spores expressing GFP at 1.2×10^7 spores/4-6 $\times 10^5$ HEK293T cells/ml for 40 min and fixed with 4% paraformaldehyde. The mean pixel intensities of RFP in an area of phagocytic cup (rectangle 2) and that of an area adjacent to the phagocytic cup in the plasma membrane (rectangle 1) were measured. The lower panel shows their relative intensities. The level of RAGE-RFP detected in the rectangle 1 was taken as 1. Scale bar, 10 µm. **g** Fluorescence intensities of RAGE-RFP were measured in phagocytic cups and plasma membrane as described in **f**, and their relative intensities are shown. Data are presented as the mean ± SEM (**a, b, d, e,** and **g**). Statistical significance was determined by two-tailed unpaired Student’s *t* tests. n=4 (**a** and **b**), n=3 (**d** and **e**), n=36 (**g**). *, $P < 0.05$; **, $P < 0.01$; ****, $P < 0.0001$; ns, not significant ($P \geq 0.05$).

Supplementary Figure 8

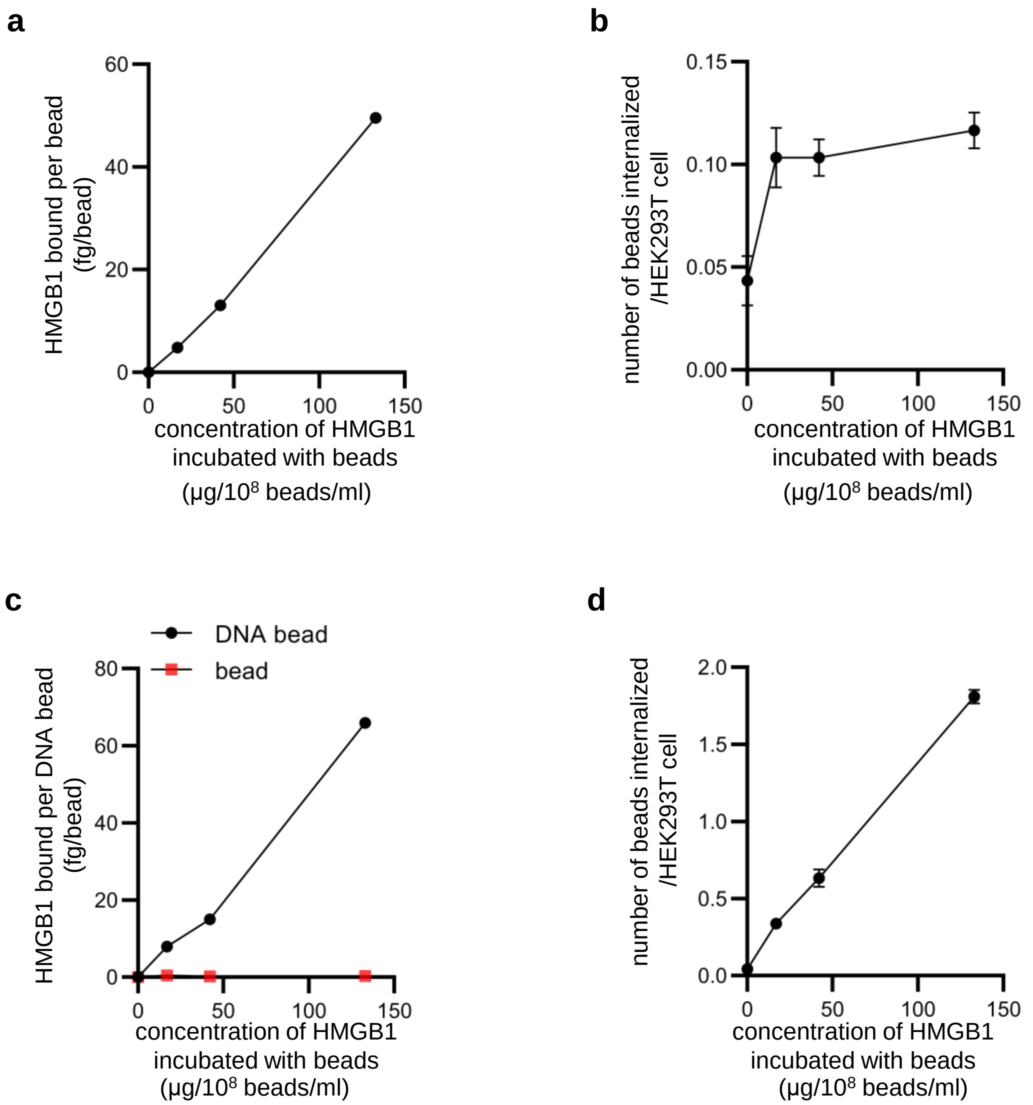

Supplementary Figure 8: HMGB1 induces phagocytosis in HEK293T cells. a Amount of HMGB1 bound to latex beads. Latex beads treated with glutaraldehyde were incubated with HMGB1 at indicated concentrations in water. Amount of HMGB1 bound to a bead were shown. **b** Internalization of HMGB1-bound latex beads in HEK293T cells. HMGB1-bound latex beads prepared as described in (**a**) were incubated with HEK293T cells. Mean numbers of beads internalized per cell are shown. **c** Amount of HMGB1 bound to latex beads or DNA beads. Latex beads or DNA beads (DNA1 was attached at 1.27 fg/bead) were incubated with HMGB1 at indicated concentrations in water. **d** Internalization of HMGB1-bound DNA beads in HEK293T cells. HMGB1-bound DNA beads prepared as described in (**c**) were incubated with HEK293T cells. Mean numbers of beads internalized per cell are shown. Data are presented as the mean ± SEM. *n*=3.

Supplementary Figure 9

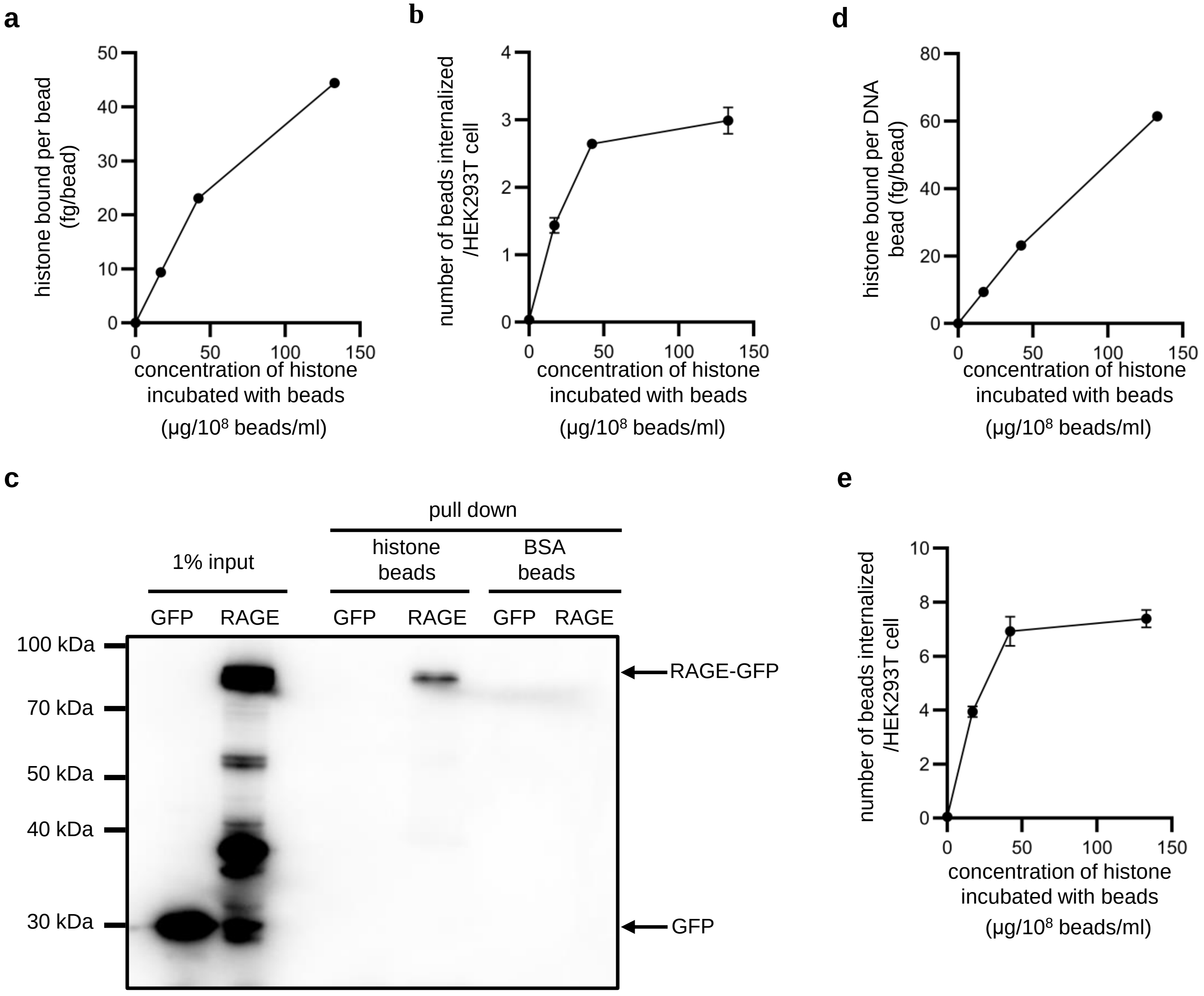

Supplementary Figure 9: Histones induce phagocytosis in HEK293T cells. a Amount of histones bound to latex beads. Latex beads treated with glutaraldehyde were incubated with indicated amount of histones. **b** Internalization of histone-bound latex beads in HEK293T cells. Histone-bound latex beads prepared as described in (**a**) were incubated with HEK293T cells. Mean numbers of beads internalized per cell are shown. **c** Analysis of interaction between RAGE and histone. HEK293T cells expressing GFP alone or RAGE-GFP were lysed and precipitated with histone beads or BSA beads. The precipitates were subjected to western blot analysis using an anti-GFP antibody. 1% of cell lysates were also subjected to western blot analysis to show proteins inputs (1% input). **d** Amount of histones bound to DNA beads. DNA beads were incubated with indicated amounts of histones. Amount of histones bound to a bead were shown. **e** Internalization of histone-bound DNA beads in HEK293T cells. Histone-bound DNA beads prepared as described in (**d**) were incubated with HEK293T cells. Mean numbers of beads internalized per cell are shown. Data are presented as the mean ± SEM (**a**, **b**, **d**, and **e**). *n*=3 (**a**, **b**, **d**, and **e**).

Supplementary Figure 10

a

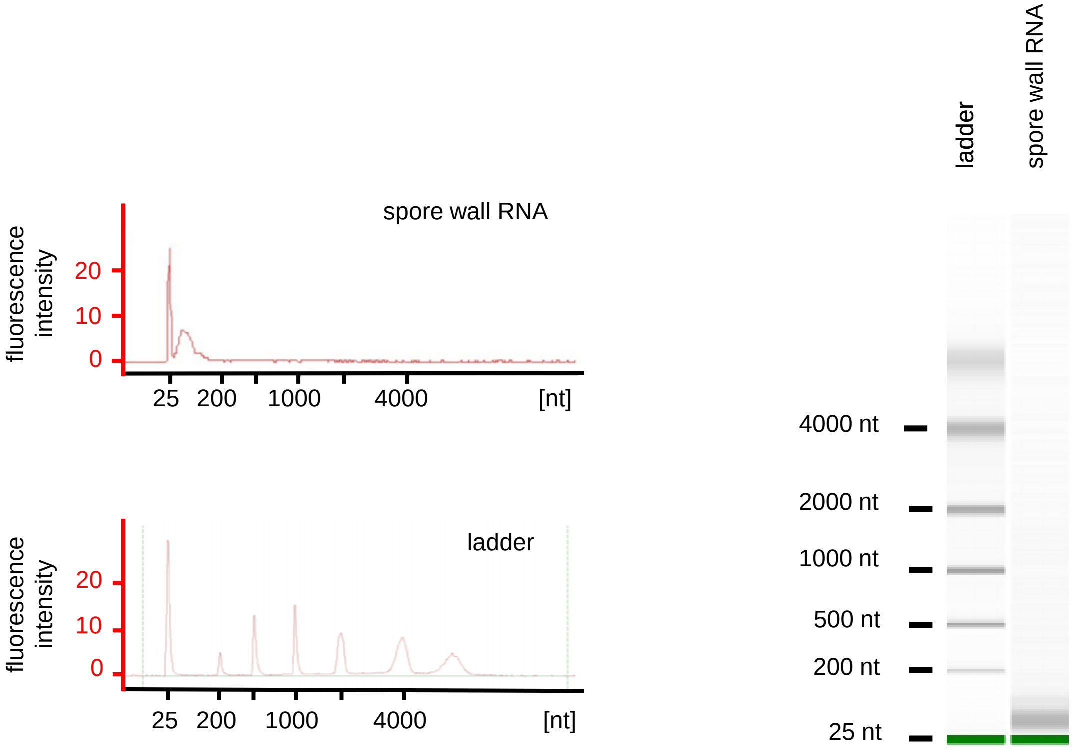

b

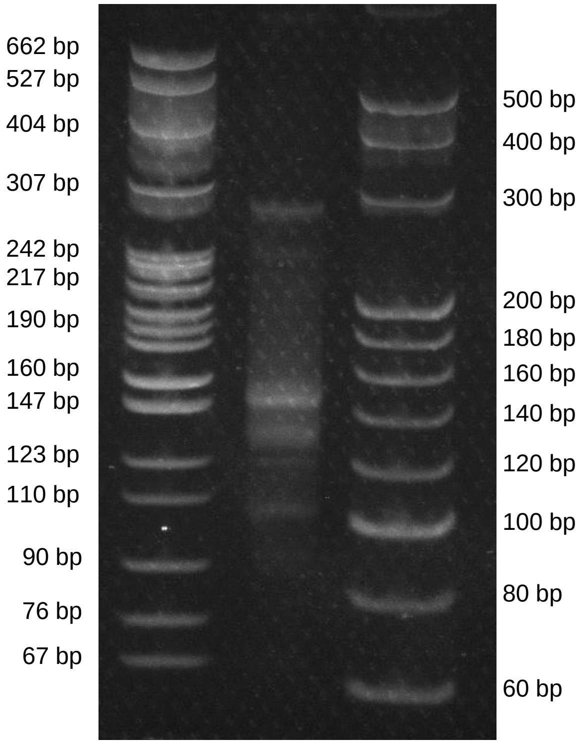

Supplementary Figure 10: Analysis of spore wall-bound RNA. a Left panels: Agilent 2100 Bioanalyzer electropherograms of spore wall RNA (top left) and single strand RNA ladder (bottom left). Right panel: Bioanalyzer gel image of RNA ladders and spore wall RNA. **b** Spore wall RNA ligated with adapters were reverse transcribed to cDNA, and PCR-amplified cDNA products were subjected to 6% polyacrylamide gel electrophoresis to separate target DNA fragments and adapters.

Supplementary Figure 11

2b

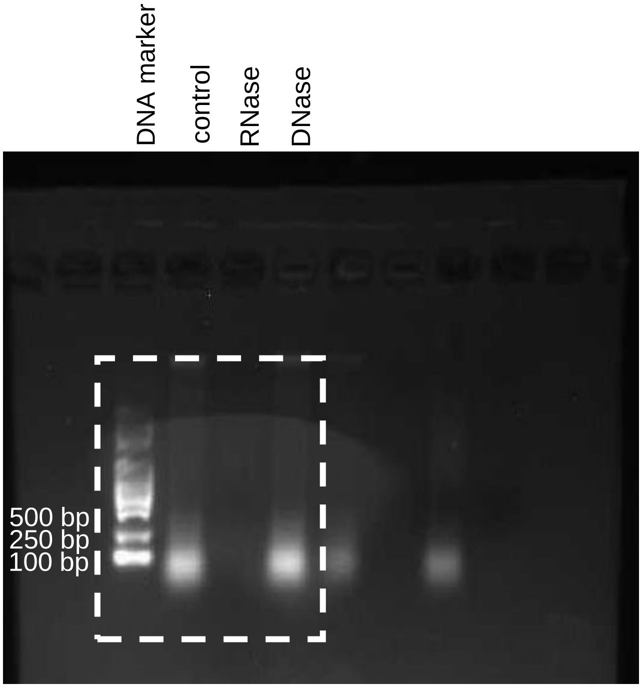

2c

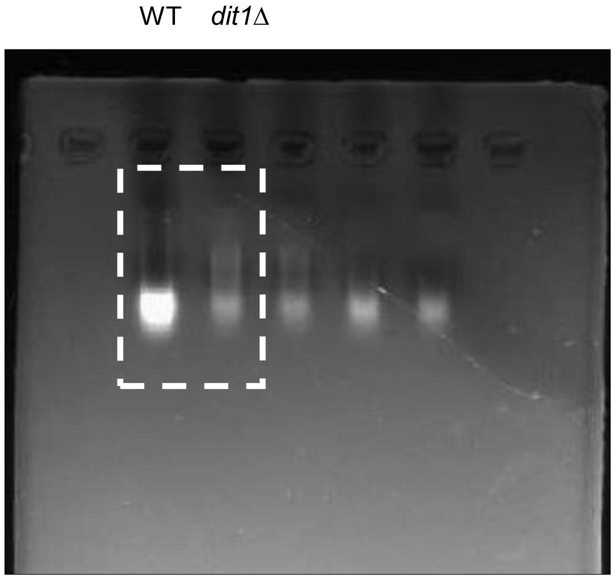

5a

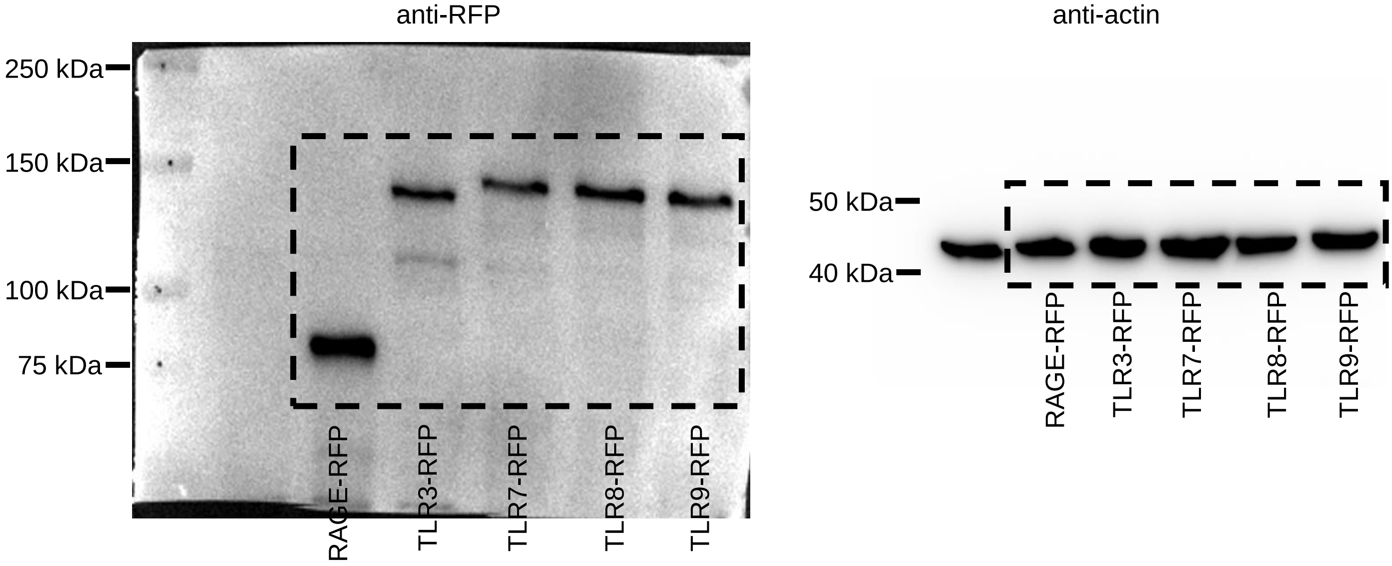

5d

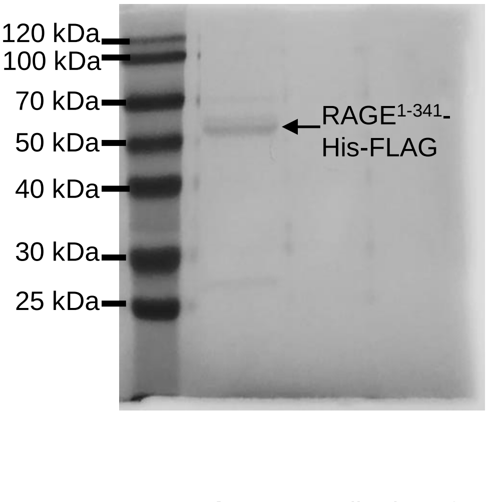

5e

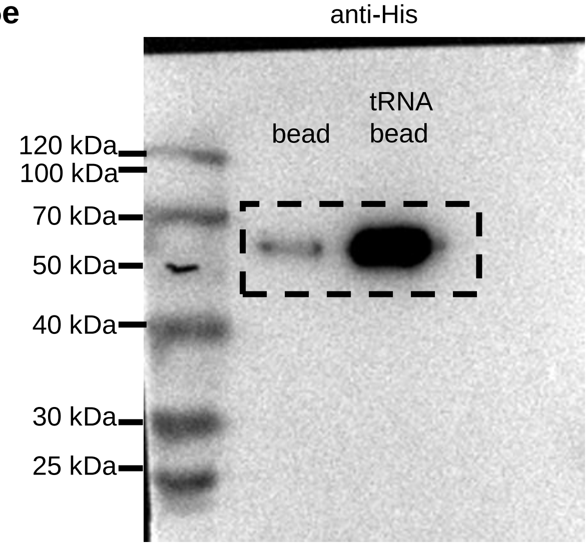

Supplementary Fig 11: Unedited gel for Fig 2 and Fig 5.

Supplementary Figure 12

S1e

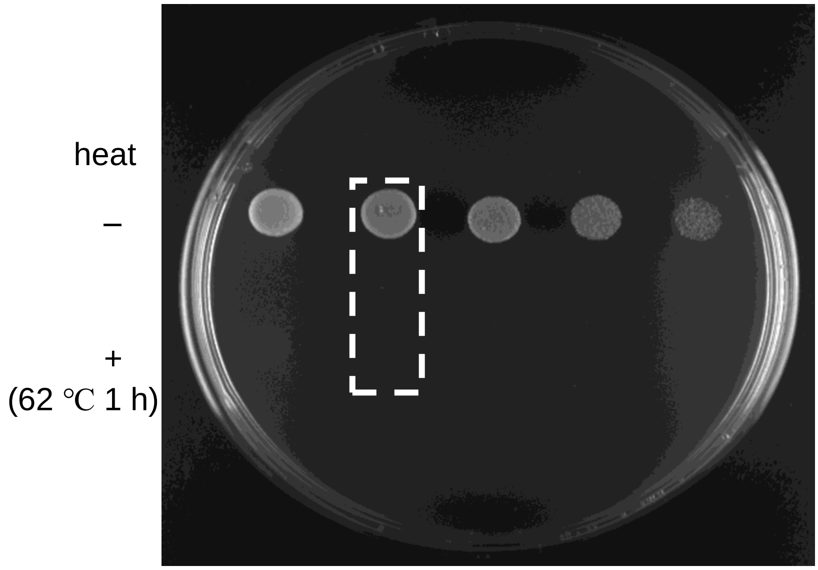

S3a

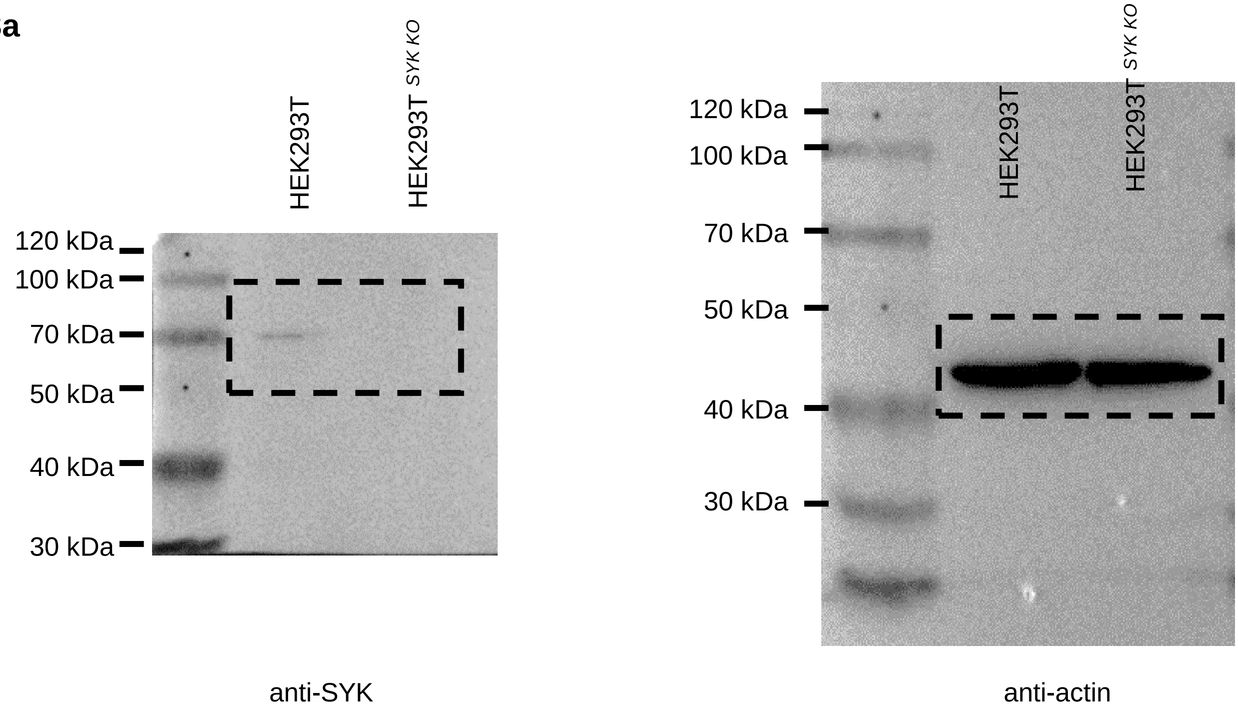

Supplementary Fig 12: Unedited spot for Supplementary Fig 1 and unedited gel for Supplementary Fig 3.

Supplementary Figure 13

S7c

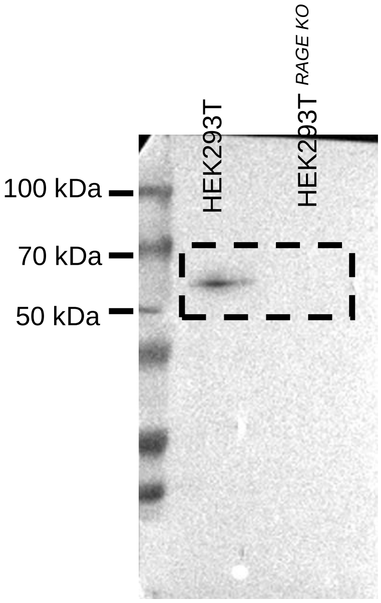

anti-RAGE

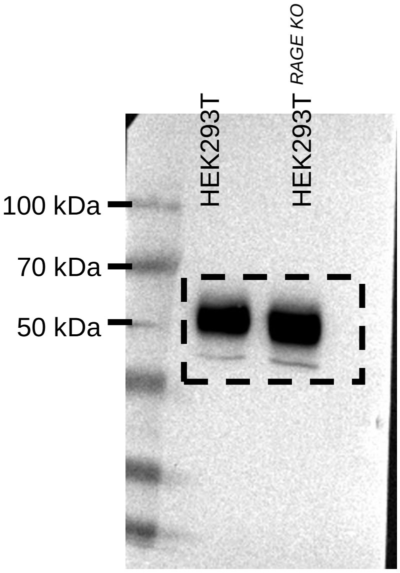

anti-GLUT1

S9c

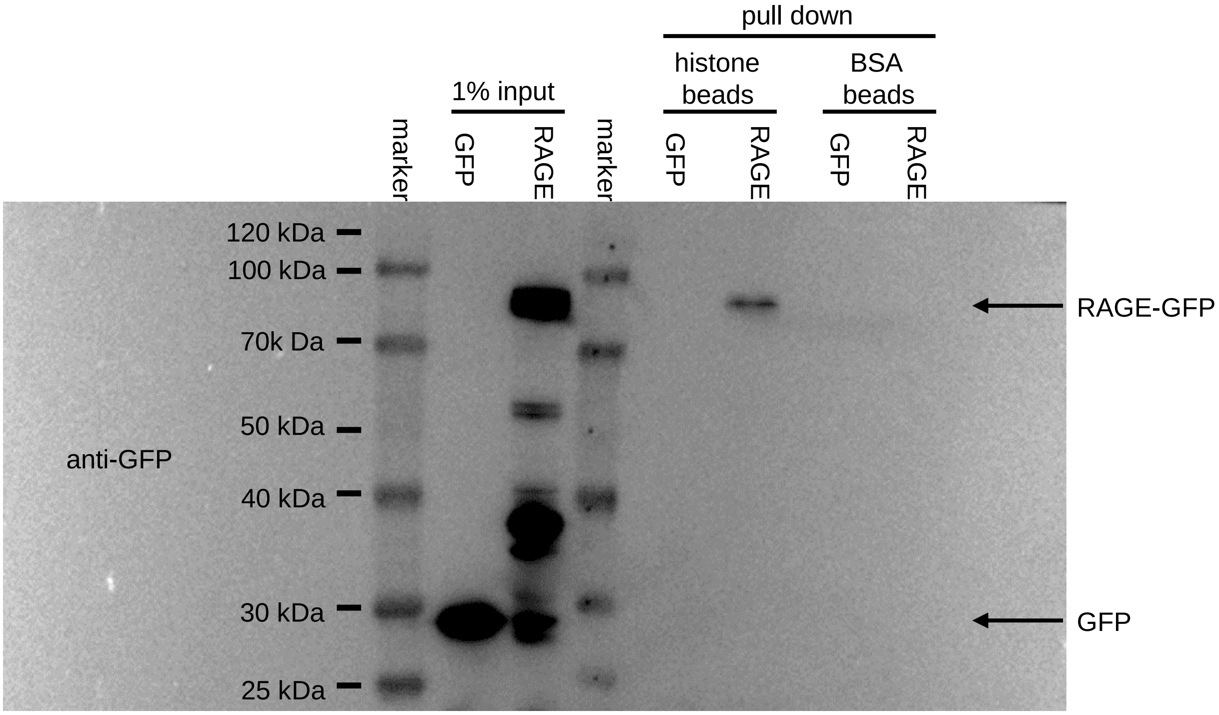

Supplementary Fig 13: Unedited gel for Supplementary Fig 7 and Fig 9..

Supplementary Table 1. Oligo nucleotides used in this study.

Name	Sequence (5'-3')	Restriction enzyme site
R1	AAAATGGAATTCATGGCTGCCGGAACAGCAGTTG	*Eco*RI
R2	CATTTTCTCGAGAGGCCCTCCAGTACTACTC	*Xho*I
R3	CAAAACATCACAGCCCGGATTG	-
R4	CAGCACCAGTGGCTCGCCAATCCGGGCTGTGATG	-
R5	CTACCGAGTCCGTGTCGCACAGATTCCTGGGGCCCCAGAAATTG	-
R6	CAATTTCTGGGGCCCCAGGAATCTGTGCGACACGGACTCGGTAG	-
R7	GGCCTTCCCCGACACGCCGCCTTGCG	-
R8	CGCAAGGCGGCGTGTCGGGGAAGGCC	-
R9	AAAATGGAATTCATGGCTGCCGGAACAGCAGTTG	-
R10	GGGTGGTTTGGCGGGGGCCCCAGCACAGGCCAGCACCAGTGGCTC	-
R11	CAATGAACAGGAATGGAGCCGAGACC	-
R12	GGTCTCGGCTCCATTCCTGTTCATTG	-
T1	AAAATGGAATTCATGGGTTTCTGCCGCAGCGC	*Eco*RI
T2	CATTTCTCGAGTTCGGCCGTGGGTCCCTGGC	*Xho*I
T3	AAAATGCAATTGATGGAAAACATGTTCCTTCAG	*Mun*I
T4	ACACACGCGTCTGCAGCATGGTGGCCTCGAGGAAATTGTATTGCTTAATG GAATCGAC	*Mlu*I
T5	AAAATGGTCGACATGGTGTTTCCAATGTGGAC	*Sal*I
T6	ACACGCGTCTGCAGCATGGTGGCCTCGACCGTTTCCTTGAACACCTG	*Mlu*I
T7	AAAATGGAATTCATGAGACAGACTTTGCCTTG	*Eco*RI
T8	CATTTTGTCGACATGTACAGAGTTTTTGGATC	*Sal*I
G1	TCCCCCGGGCTGCAGATGAGTAAAGGAGAAGAACTT	*Pst*I
G2	TTACATGACTCGAGTCATTTGTATAGTTCATCCATGCC	*Xho*I
S1	AAATGCTCGAGATGGCTGCCGGAACAGCAGTTG	*Xho*I
S2	CATTTACGCGTGTGGTGGTGGTGGTGGTGGCCTGCTAGAGTTCCCAGCC CTGATC	*Mlu*I
H1	CTGCGGAATTCATGGCTGCCGGAACAGC	*Eco*RI
H2	CTAGCGGCCGCCTAGTGGTGGTGGTGGTGGTGGCCTGCCCCGGCTCCT GCAGGCCCTCCAGTACTACTCTCG	*Not*I
SG1	AAAAGAATTCATGGCCAGCAGCGGCATGAC	*Eco*RI
SG2	AAAAACGCGTGTTCACCACGTCATAGTAGT	*Mlu*I
Rg1	CACCCAAGAAACCACCCCAGCGGC	*Bpi*I
Rg2	AAACGCCGCTGGGGTGGTTTCTTG	*Bpi*I
Rg3	CACCTCTTACGGTAGACACGGACT	*Bpi*I
Rg4	AAACAGTCCGTGTCTACCGTAAGA	*Bpi*I
Tg1	CACCGTACCTGAGTCAACTTCAGG	*Bpi*I
Tg2	AAACCCTGAAGTTGACTCAGGTAC	*Bpi*I
Tg3	CACCGCCTTGTATCTACTTTTGGG	*Bpi*I
Tg4	AAACCCCAAAAGTAGATACAAGGC	*Bpi*I
Sg1	CACCGGCAGAAGATTACCTGGTCC	*Bpi*I
Sg2	AAACGGACCAGGTAATCTTCTGCC	*Bpi*I
Sg3	CACCGCTTCTTGAGGAGGCAGACC	*Bpi*I
Sg4	AAACGGTCTGCCTCCTCAAGAAGC	*Bpi*I
M-qGAPDH F	CATCACTGCCACCCAGAAGACTG	-
M-qGAPDH R	ATGCCAGTGAGCTTCCCGTTCAG	-
M-qRAGE-F	GCCACTGGAATTGTCGATGAGG	-
M-qRAGE-R	GCTGTGAGTTCAGAGGCAGGAT	-
H-qRAGE F	CACCTTCTCCTGTAGCTTCAGC	-
H-qRAGE R	AGGAGCTACTGCTCCACCTTCT	-
H-qGAPDH F	AGATCATCAGCAATGCCTCCTG	-
H-qGAPDH R	ATGGCATGGACTGTGGTCATG	-
RAGE check-F1	GTAGTAGGTGCTCAAAACATC	-
RAGE check-R1	GGAGGCCTTGGAGAAGACCC	-
TLR3 check-F1	GCTATTAATGTTGCTCATAC	-
TLR3 check-R1	GTAAGGTTCAACACTGTTATG	-
SYK check-F1	GAAGCATGGCCAGCAGCGGC	-
SYK check-R1	CTTCACATATTCCCTGATGAG	-
DNA1	GTGCCAGATCGGGGTTCAATTC	-
DNA2	AGGGCACTCACCGTGGAGATGA	-
cy3-RNA	GUGCCAGAUCGGGGUUCAAUUC	-

Supplementary Table 2. Plasmids used in this study.

Name	Parental plasmid	Description	Cloning sites
pME-RAGE-tagRFP	pME-tagRFP	Gene, RAGE; Primers, R1 and R2	*Eco*RI/*Xho*I
pME-RAGE-mEGFP	pME-mEGFP	Gene, RAGE from pME-RAGE-tagRFP	*Eco*RI/*Xho*I
pME-RAGE^{mut1}-tagRFP	pME-tagRFP	pME-RAGE-tagRFP was mutated with R9, R10, R11, and R12	*Eco*RI/*Xho*I
pME-RAGE^{mut2}-tagRFP	pME-tagRFP	pME-RAGE-tagRFP was mutated with R3, R4, R5, R6, R7, and R8	*Eco*RI/*Xho*I
pME-SYK-mEGFP	pME-mEGFP	Gene, SYK; Primers, SG1 and SG2	*Eco*RI/*Mlu*I
pME-TLR3-tagRFP	pME-tagRFP	Gene, TLR3; Primers, T7 and T8	*Eco*RI/*Xho*I
pME-TLR7-tagRFP	pME-tagRFP	Gene, TLR7; Primers, T5 and T6	*Xho*I/*Mlu*I
pME-TLR8-tagRFP	pME-tagRFP	Gene, TLR8; Primers, T3 and T4	*Eco*RI/*Mlu*I
pME-TLR9-tagRFP	pME-tagRFP	Gene, TLR9; Primers, T1 and T2	*Eco*RI/*Xho*I
pME-sRAGE-His-Flag	pME-His-Flag	Gene, sRAGE; Primers, S1 and S2	*Xho*I/*Mlu*I
pME-RAGE-His	pME-His-Flag	Gene, RAGE; Primers, H1 and H2	*Eco*RI/*Not*I
pX330EGFP-hU6-RAGE gRNA1-hSpCas9	pX330EGFP-hU6-gRNA-hSpCas9	Gene, CAAGAAACCACCCCAGCGGC; Primers, Rg1 and Rg2	*Bpi*I
pX330EGFP-hU6-RAGE gRNA2-hSpCas9	pX330EGFP-hU6-gRNA-hSpCas9	Gene, TCTTACGGTAGACACGGACT; Primers, Rg3 and Rg4	*Bpi*I
pX330EGFP-hU6-TLR3 gRNA1-hSpCas9	pX330EGFP-hU6-gRNA-hSpCas9	Gene, GTACCTGAGTCAACTTCAGG; Primers, Tg1 and Tg2	*Bpi*I
pX330EGFP-hU6-TLR3 gRNA2-hSpCas9	pX330EGFP-hU6-gRNA-hSpCas9	Gene, GCCTTGTATCTACTTTTGGG; Primers, Tg3 and Tg4	*Bpi*I
pX330EGFP-hU6-SYK gRNA1-hSpCas9	pX330EGFP-hU6-gRNA-hSpCas9	Gene, GGCAGAAGATTACCTGGTCC; Primers, Sg1 and Sg2	*Bpi*I
pX330EGFP-hU6-SYK gRNA2-hSpCas9	pX330EGFP-hU6-gRNA-hSpCas9	Gene, GCTTCTTGAGGAGGCAGACC; Primers, Sg3 and Sg4	*Bpi*I
pLIB2-mEGFP-BSD	pLIB2 -BSD	Gene, mEGFP from pME-mEGFP	*Eco*RI/*Not*I
pGP	pGP	For retrovirus transfection	-
pLC-VSVG	pLC-VSVG	For retrovirus transfection	-
pRS306-TEF-GFP	pRS306	Gene, GFP; Primers, G1 and G2; The TEF2 promoter and CYC1 terminator were digested out of pRS316TEF	GFP, *Pst*I/*Xho*I; TEF2 promoter, *Sac*I/*Pst*I; CYC1 terminator, *Xho*I/*Kpn*I
