# Supplementary material for: Receptor for advanced glycation end-products (RAGE) mediates phagocytosis in nonprofessional phagocytes
Source: Commun Biol. 2022 Aug 16;5:824. doi: 10.1038/s42003-022-03791-1 (PMC9381800; doi:10.1038/s42003-022-03791-1)
Supplement: Supplementary file 3 — Description of Additional Supplementary Files [file 42003_2022_3791_MOESM3_ESM.pdf]

Description of Additional Supplementary Files

File name: Supplementary Data 1
Description: The source data behind the graphs in the paper have been uploaded as Supplementary Data in the Excel format.

File name: Supplementary Video 1
Description: Phagocytic internalization of a spore by a HEK293T cell**.** HEK293T cells transiently expressing RAGE-GFP were preincubated with LysoTracker Red. After changing the medium, the cells were incubated with spores. The internalization process was followed by time-lapse microscopy at 1-min intervals.
