# Supplementary material for: Receptor for advanced glycation end-products (RAGE) mediates phagocytosis in nonprofessional phagocytes
Source: Commun Biol. 2022 Aug 16;5:824. doi: 10.1038/s42003-022-03791-1 (PMC9381800; doi:10.1038/s42003-022-03791-1)
Supplement: Supplementary file 6 — Reporting Summary [file 42003_2022_3791_MOESM6_ESM.pdf]

nature research

Corresponding author(s): *Double-blind peer review submissions: write DBPR and your manuscript number here instead of author names.*

Last updated by author(s): *YYYY-MM-DD*

Reporting Summary

Nature Research wishes to improve the reproducibility of the work that we publish. This form provides structure for consistency and transparency in reporting. For further information on Nature Research policies, see our Editorial Policies and the Editorial Policy Checklist.

Statistics

For all statistical analyses, confirm that the following items are present in the figure legend, table legend, main text, or Methods section.

n/a	Confirmed	
☐	☒	The exact sample size (n) for each experimental group/condition, given as a discrete number and unit of measurement
☐	☒	A statement on whether measurements were taken from distinct samples or whether the same sample was measured repeatedly
☐	☒	The statistical test(s) used AND whether they are one- or two-sided<br>*Only common tests should be described solely by name; describe more complex techniques in the Methods section.*
☒	☐	A description of all covariates tested
☐	☒	A description of any assumptions or corrections, such as tests of normality and adjustment for multiple comparisons
☐	☒	A full description of the statistical parameters including central tendency (e.g. means) or other basic estimates (e.g. regression coefficient) AND variation (e.g. standard deviation) or associated estimates of uncertainty (e.g. confidence intervals)
☐	☒	For null hypothesis testing, the test statistic (e.g. F, t, r) with confidence intervals, effect sizes, degrees of freedom and P value noted<br>*Give P values as exact values whenever suitable.*
☒	☐	For Bayesian analysis, information on the choice of priors and Markov chain Monte Carlo settings
☒	☐	For hierarchical and complex designs, identification of the appropriate level for tests and full reporting of outcomes
☒	☐	Estimates of effect sizes (e.g. Cohen's d, Pearson's r), indicating how they were calculated

Our web collection on statistics for biologists contains articles on many of the points above.

Software and code

Policy information about availability of computer code

Data collection	Flow cytometric data were collected using BD FACS AriaIII by AriaIII software.<br>Western blotting image were obtained by Tannon 5200 Multi.<br>Agarose gel electrophoresis image were obtained by Bio-Rad Universal Hood II Gel Dox XR System.<br>Microscopy images were obtained using a Nikon C2 Eclipse Ti-E inverted microscope with a DS-Ri camera (live imaging and yeast images) or a Nikon C2 Eclipse Ti-E inverted confocal laser scanning microscope (the other images) equipped with NIS-Element AR software.
Data analysis	Flow cytometric data were analyzed by BD FlowJo 7.6.<br>RFP fluorescence intensity were analyzed by NIS-Element AR software.<br>Statistical analysis were performed using GraphPad Prism 8 and Microsoft Excel 2016. t-test was employed to determine similarities between tow groups. *, P < 0.05; **, P < 0.01; ***, P < 0.001; ****, P < 0.0001. Data were combined from at least three independent experiments unless otherwisestated.

For manuscripts utilizing custom algorithms or software that are central to the research but not yet described in published literature, software must be made available to editors and reviewers. We strongly encourage code deposition in a community repository (e.g. GitHub). See the Nature Research guidelines for submitting code & software for further information.

Data

Policy information about availability of data

All manuscripts must include a data availability statement. This statement should provide the following information, where applicable:
- Accession codes, unique identifiers, or web links for publicly available datasets
- A list of figures that have associated raw data
- A description of any restrictions on data availability

All the data relevant materials including primers, reagents and cells within the Article or Supplementary information. The data used to support the finding of this study are available from the corresponding author upon reasonable request. The RNA sequencing result was deposited in the Sequence Read Archive database, accession number PRJNA748575.

Field-specific reporting

Please select the one below that is the best fit for your research. If you are not sure, read the appropriate sections before making your selection.

☒ Life sciences ☐ Behavioural & social sciences ☐ Ecological, evolutionary & environmental sciences

For a reference copy of the document with all sections, see nature.com/documents/nr-reporting-summary-flat.pdf

Life sciences study design

All studies must disclose on these points even when the disclosure is negative.

Sample size	The exact sample size is given in the legend of each figure, and each experiment was performed in three parallel experiments. The representative results are shown.
Data exclusions	No data was excluded.
Replication	All of experiments were performed at least three times. Experimental results are reliably reproduced.
Randomization	For Microscope analysis phagocytosis experiments, randomly selected cells to analyze internalization efficiency.
Blinding	All samples were exposed to the same experimental conditions. Therefore, no blinding was necessary.

Reporting for specific materials, systems and methods

We require information from authors about some types of materials, experimental systems and methods used in many studies. Here, indicate whether each material, system or method listed is relevant to your study. If you are not sure if a list item applies to your research, read the appropriate section before selecting a response.

Materials & experimental systems

n/a	Involved in the study	
☐	☒	Antibodies
☐	☒	Eukaryotic cell lines
☒	☐	Palaeontology and archaeology
☒	☐	Animals and other organisms
☒	☐	Human research participants
☒	☐	Clinical data
☒	☐	Dual use research of concern

Methods

n/a	Involved in the study	
☒	☐	ChIP-seq
☐	☒	Flow cytometry
☒	☐	MRI-based neuroimaging

Antibodies

Antibodies used	Primary Antibodies: anti-RFP (RF5R,InvivoGen), anti-RAGE (ab216329, abcam), anti-SYK (ab40781),anti-GLUT1 (ab115730, abcam), anti-GFP (HT801, TransGen Biotech,), anti-actin (HC201, TransGen Biotech), anti-His (HT501, TransGen Biotech)<br>Secondary Antibodies: anti-mouse IgG HRP-linked (HS201,TransGen Biotech), anti-rabbit IgG HRP-linked (HS101-01,TransGen Biotech),
Validation	All antibodies were validated by the manufactures or were validated in the previous publications.<br>The website for some used antibodies.<br>https://www.transgenbiotech.com//index.php?c=index&a=search&kw=HT801<br>https://www.transgenbiotech.com//index.php?c=index&a=search&kw=HC201<br>https://www.transgenbiotech.com//index.php?c=index&a=search&kw=HS201<br>https://www.transgenbiotech.com//index.php?c=index&a=search&kw=HS101-01

https://www.transgenbiotech.com//index.php?c=index&a=search&kw=HT501
https://www.abcam.com/syk-antibody-ep573y-ab40781.html
https://www.thermofisher.cn/antibody/primary/query/rfp
https://www.abcam.com/glucose-transporter-glut1-antibody-epr3915-ab115730.html
https://www.abcam.com/rage-antibody-epr21171-ab216329.html

Eukaryotic cell lines

Policy information about cell lines

Cell line source(s)	HEK293 cell line (ATCC CRL-1573), HEK293T cell line (ATCC, CRL-11268), HUC cell line (ATCC, CRL-9520™), HeLa cell line (ATCC, CRM-CCL-2), Jurkat cell line (ATCC, TIB-152). Primary mouse ATII cells (CP-M003; Procell life Science & Technology Co,.Ltd, Wuhan, China).
Authentication	The cell lines were authenticated by the respective company.
Mycoplasma contamination	Original parental HEK293 HEK293T HUC and HeLa cell lines was confirmed as negative for mycoplasma contamination. The knockout cell lines derived from HEK293T cells were not tested for mycoplasma contamination.
Commonly misidentified lines (See ICLAC register)	None of the cell lines used are listed in the ICLAC database.

Flow Cytometry

Plots

Confirm that:

☒ The axis labels state the marker and fluorochrome used (e.g. CD4-FITC).

☒ The axis scales are clearly visible. Include numbers along axes only for bottom left plot of group (a 'group' is an analysis of identical markers).

☒ All plots are contour plots with outliers or pseudocolor plots.

☒ A numerical value for number of cells or percentage (with statistics) is provided.

Methodology

Sample preparation	Cells (2 X 10^5/well) were seeded in 12-well plates and allowed to grow to a density 4–6 X 10^5cells /well. The cells were incubated with 1.2 x 10^7 spores expressing GFP in a CO2 incubator at 37°C for 1 h. Then, the cells were treated with trypsin for 5 min and washed twice with PBS. The samples were analyzed by BD FACS AriaIII (BD).
Instrument	BD FACS AriaIII
Software	BD FACS AriaIII software was used to collect data; and the data were analyzed using FlowJo X (BD).
Cell population abundance	We only used cultured cell lines, so cell population abundane was always 100%. At least 10000 cells were analyzed for each sample.
Gating strategy	We only used cell lines and gating of different populations does not apply to this study.

☐ Tick this box to confirm that a figure exemplifying the gating strategy is provided in the Supplementary Information.
